# *SPRY4* suppresses proliferation and induces apoptosis of colorectal cancer cells by repressing oncogene *EZH2*

**DOI:** 10.18632/aging.202859

**Published:** 2021-04-20

**Authors:** Jia Guo, Huadong Zhu, Qiang Li, Jianhua Dong, Wei Xiong, Kun Yu

**Affiliations:** 1 Department of Gastroenterology, Sunshine Union Hospital, Weifang 261000, China; 2 School of Life Science, Nanchang University, Nanchang 330031, China; 3 Department of Colorectal Cancer Surgery, Yunnan Cancer Hospital, The Third Affiliated Hospital of Kunming Medical University, Kunming 650118, China

**Keywords:** colorectal cancer, SPRY4, EZH2, cell proliferation

## Abstract

Colorectal cancer (CRC), a common malignant tumor in the digestive tract, is a leading cause of cancer-related death. *SPRY4* has been reported to act as a tumor suppressor gene in various tumors. This study aims to assess the role of SPRY4 in colorectal cancer (CRC) and uncover its underlying mechanisms. Firstly, the expression levels of *SPRY4* were measured in CRC cell lines. *SPRY4*-overexpressing or silencing plasmids were transfected into CRC cells to regulate its expression level. CCK-8, colony formation, EdU assay, wound-healing and Transwell assays were performed to determine cell proliferation, invasion and migration abilities. Then, apoptosis was measured by flow cytometry analysis, and the expression of apoptosis-related protein was analyzed by western-blotting. Next, the *in vivo* tumorigenesis assay was performed in nude mice. According to the results, there was a lower expression of *SPRY4* in CRC cell lines compared with normal cell line, and the overexpression of *SPRY4* significantly suppressed cell proliferation, migration and invasion, and promoted apoptosis in SW480 cells. Moreover, the enhanced proliferation, invasion and migration upon *SPRY4* silencing was reversed by *EZH2* inhibition. In addition, we found that the overexpression of *SPRY4* inhibited tumorigenesis *in vivo* by diminishing the size and weight of the tumors. Our study indicates that *SPRY4* might be a potential tumor suppressor gene and prognostic factor for patients with CRC.

## INTRODUCTION

Despite significant progress in therapeutic strategies has been achieved in recent years, colorectal cancer (CRC) still ranks the third most frequent tumor worldwide, followed by lung and breast carcinoma [1]. The mortality of CRC in developed countries is high, and is keeping an increasing tendency [2]. At present, there are over 1 million newly diagnosed cases each year, seriously threatening People’s health and quality of life [3]. Therefore, in-depth exploration of the process and specific molecular mechanism of CRC is particularly important, which may offer the references or strategies for updated treatments.

Sprouty (SPRY) are initially identified as the inhibitors of receptor tyrosine kinase regulating tracheal branching in *Drosophila* [4]. *SPRY4*, a membrane of SPRY family (*SPRY1-4*), is generally recognized as a regulator of receptor tyrosine kinases [5]. In addition, *SPRY4* also exerts regulatory effects on cell growth, differentiation, and metastasis during the development of malignant tumors in various types of cancers [6, 7]. *SPRY4* suppresses the migration and stem cell-related properties of breast carcinoma cells and suppresses cell motility of prostate cancer cells [8–10]. Nevertheless, *SPRY4* acts as an oncogene to promote ovarian cancer invasion and accelerate human ovarian cancer progression [11]. Thus, *SPRY4* plays a dual role in human cancers and the function of *SPRY4* often varies among different tumor types. However, up to date, the role of *SPRY4* in CRC still remains unclear.

*EZH2*, a histone-lysine N-methyltransferase enzyme, has been documented to be associated with the occurrence and development of malignant tumors [12–14], and also be involved in the cellular biological functions of various cancers, including gastric cancer, hepatocellular carcinoma, bladder cancer, and nasopharyngeal cancer [15–18]. Furthermore, several studies have reported the abnormally high expression of *EZH2*, which is also positively correlated with the poor prognosis of CRC [19–21], indicating an important role of *EZH2* in CRC.

The aim of the present study is to explore the role of *SPRY4* during tumor progression of CRC *in vivo* and *in vitro*. In addition, this study also investigates the relationship between *SPRY4* and *EZH2* to further understand the potential regulatory mechanism.

## RESULTS

### Differential expression of *SPRY4* and *EZH2* between colorectal cancer cells and normal human colon cell

**Figure 1:**
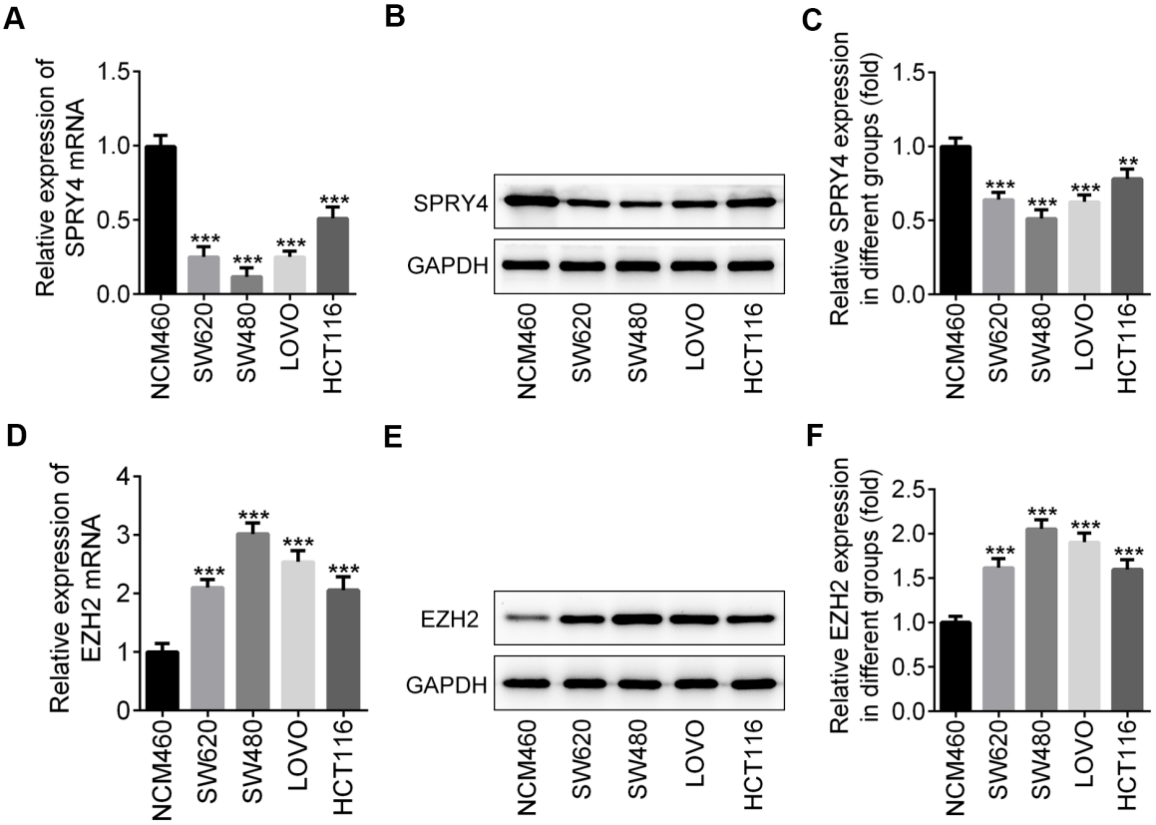
**Differential expression of *SPRY4* and *EZH2* between colorectal cancer cells and normal human colon cell.** From four human CRC cell lines, SW620, SW480, LOVO and HCT116, and a normal human colon mucosal epithelial cell line NCM460, we detected the mRNA level and protein expression of *SPRY4* using western blotting and RT-qPCR, respectively (**A**–**C**). The mRNA level and protein expression of EZH2 were detected using western blotting and RT-qPCR, respectively (**D**–**F**). **, ***p<0.01, 0.001 vs NCM460.

To explore the role of *SPRY4* in CRC, we first screened the expression level of *SPRY4* and *EZH2* in four human CRC cell lines, SW620, SW480, LOVO and HCT116, and a normal human colorectal epithelial cell line, NCM460, via western blotting and RT-qPCR. As shown in Figure 1A–1C, the translation and transcription levels of *SPRY4* genes between normal cell line and cancer cell lines were statistically different. In particular, NCM460 cell line exhibited the highest level of *SPRY4*, while SW480 exhibited the lowest. In contrast, the expression of *EZH2* gene was upregulated in all cancer cell lines, and SW480 showed the highest level (Figure 1D–1F). Therefore, we selected SW480 cell line to continue the investigation of *SPRY4*.

### Effects of *SPRY4* overexpression on CRC cell proliferation

**Figure 2:**
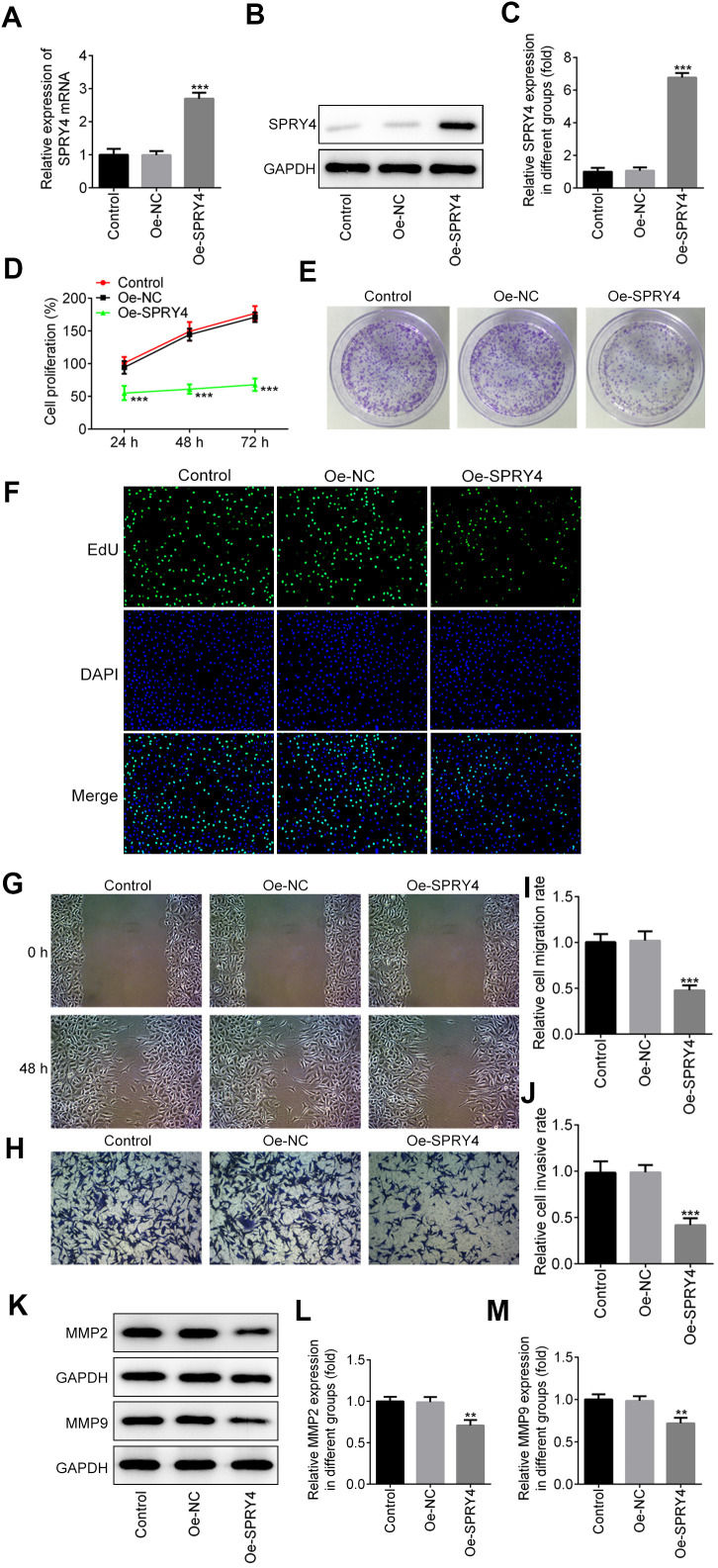
**Effects of *SPRY4* overexpression on CRC cell proliferation, migration and invasion.** The overexpression plasmid of *SPRY4* was constructed and transfected into SW480 cells. The mRNA level and protein expression of *SPRY4* were detected using RT-qPCR and western blotting, respectively (**A**–**C**). Cell proliferation ability was determined using CCK-8 assay (**D**), cell colony formation (**E**), and EdU assay (**F**). Wound-healing and Transwell assays were conducted to measure cell migration and invasion, respectively (**G**–**J**). Protein expression of MMP2 and MMP9 was detected by western blotting (**K**–**M**). **, ***p<0.01, 0.001 vs Oe-NC.

To examine the impacts of *SPRY4*, we constructed the overexpression plasmid of SPRY4 and the enhanced expression of *SPRY4* was observed in transfected SW480 (Figure 2A–2C). Next, the effects of *SPRY4* overexpression on SW480 cell proliferation were determined by CCK8, colony formation and EdU assays. As shown in Figure 2D, *SPRY4* overexpression dramatically suppressed the proliferation ability of SW480 cells in a time-dependent manner. Additionally, compared with negative control group, the number of cell colonies was also significantly reduced upon *SPRY4* overexpression (Figure 2E). Furthermore, EdU assay further revealed that overexpression of *SPRY4* obviously decreased the proliferation ability of SW480 cells (Figure 2F).

### Effects of *SPRY4* overexpression on invasion and migration of CRC cell

We further evaluated the impacts of *SPRY4* on CRC cell mobility. As exhibited by the *in vitro* scratch assay (Figure 2G, 2I), wound closure of SW480 cell was decreased upon *SPRY4* overexpression, compared to the control. For the Transwell assay, SW480 cells with different treatments were allowed to invade for 48 h and a similar trend was also observed. The *SPRY4* overexpression treatment resulted in the least amount of cancer cells in the lower chamber (Figure 2H, 2J). Since matrix metalloproteinase 2 (MMP2) and MMP9 are the key members of the MMP family which can degrade the extracellular matrix, we continued to evaluate their expression mediated by *SPRY4* overexpression with western-blotting assay. As depicted in Figure 2K–2M, the expression of MMP2 and MMP9 was downregulated by *SPRY4*, which was only about half of that of control group. In general, these data indicated a preventive role of *SPRY4* against invasion and migration of CRC cells.

### Effect of *SPRY4* overexpression on apoptosis in CRC cells

**Figure 3:**
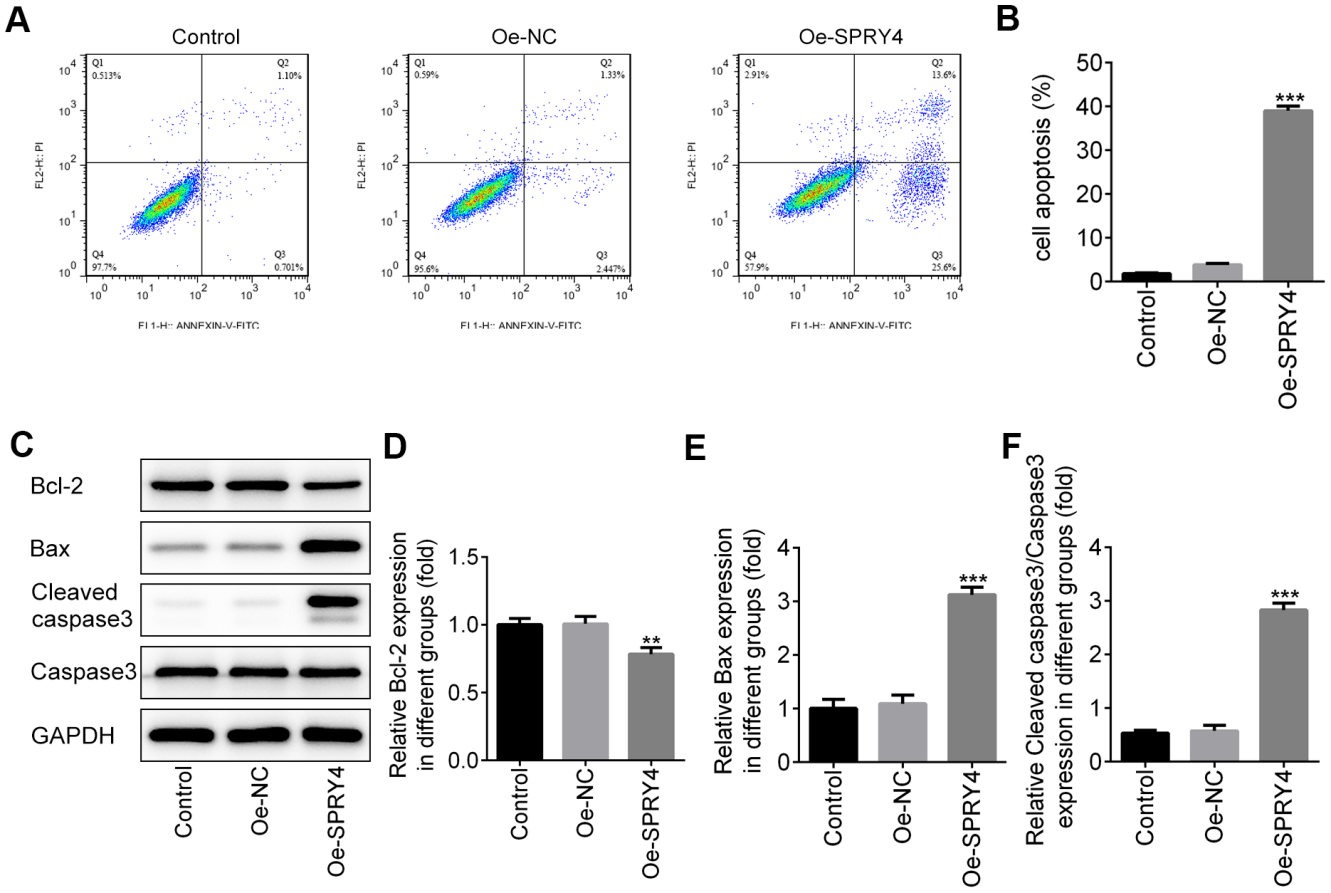
**Effects of *SPRY4* overexpression on CRC cell apoptosis.** The overexpression plasmid of *SPRY4* was constructed and transfected into SW480 cells. Then, cell apoptotic rate was analyzed using flow cytometry assay (**A**, **B**). Furthermore, the protein expressions of Bcl-2, Bax, Cleaved caspase-3 and caspase-3 were measured using western blotting (**C**–**F**). **, ***p<0.01, 0.001 vs Oe-NC.

Next, we assessed the impacts of *SPRY4* on apoptosis in SW480 cells. As shown in Figure 3A, 3B, compared to the control group, *SPRY4* overexpression remarkably improved cell apoptosis rate. In addition, *SPRY4* overexpression upregulated the protein expressions of pro-apoptosis-related Bax and cleaved caspase-3 while down-regulated the anti-apoptosis-related Bcl-2 (Figure 3C–3F), which further demonstrated the promotive effect on cell apoptosis in SW480 cells.

### *SPRY4* reduced the expression of EZH2 and MDM2

**Figure 4:**
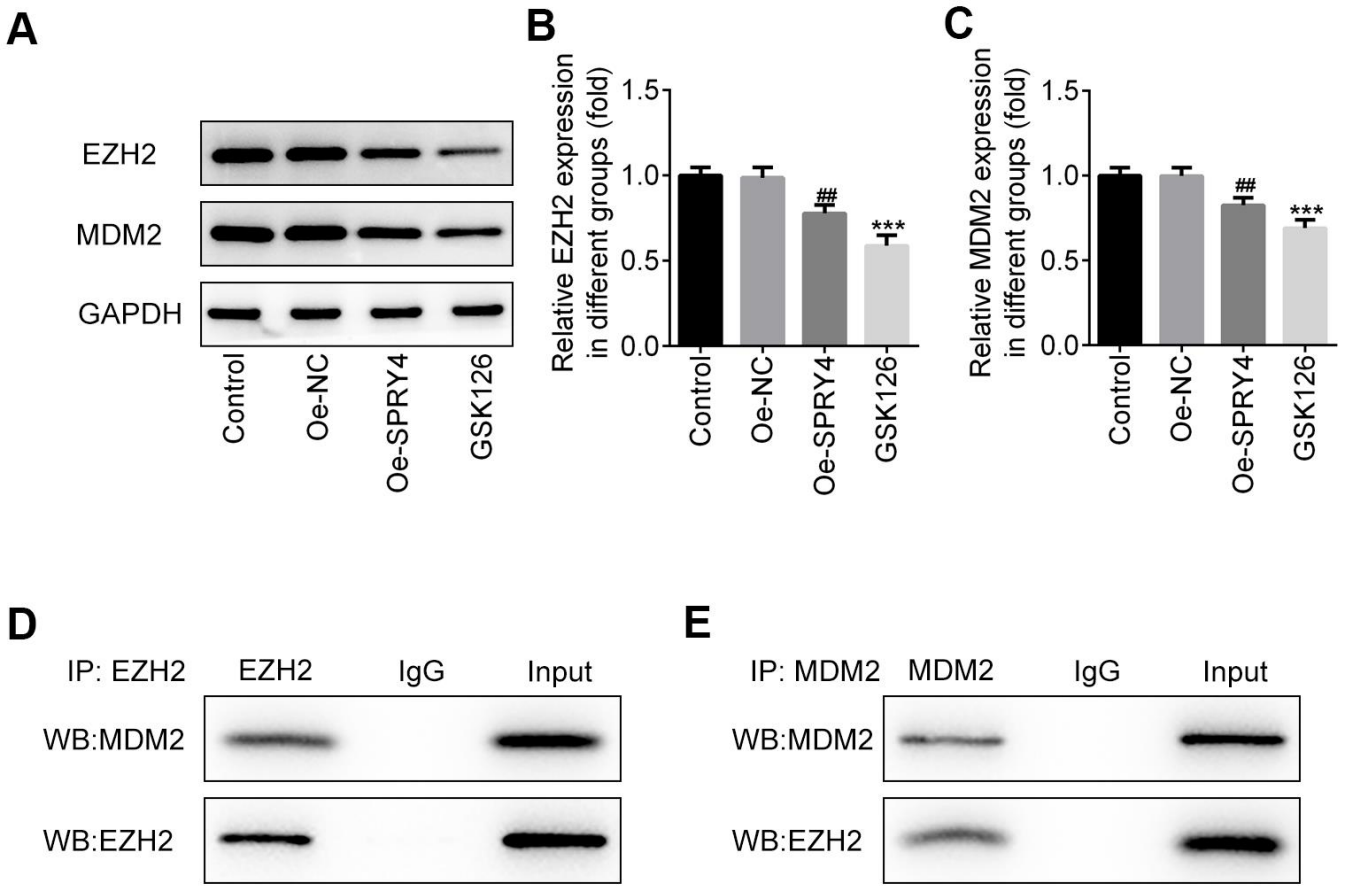
***SPRY4* reduced the expression of EZH2 and MDM2.** SW480 cells were transfected with overexpression plasmid of *SPRY4* or co-treated with overexpression plasmid of *SPRY4* and GSK126 (an EZH2 inhibitor). In each group, protein expressions of EZH2 and MDM2 were detected using western blotting (**A**–**C**). Immunoprecipitation assay, followed by western blotting, was used to evaluate the association between EZH2 and MDM2 (**D**, **E**). ***p<0.001 vs Control; ##p<0.01 vs Oe-NC.

SW480 cells is a p53 double-mutant cell line, whereas the main target of MDM2 is wild-type p53 [22]. In addition, activated p53 was demonstrated to suppress EZH2 gene expression through repression of the EZH2 gene promoter, and SPRY4 was reported to promote tumor cell proliferation by activating EZH2 [23, 24], thus, the relationship among SPRY4, MDM2 and EZH2 in SW480 cells aroused our interest. Intriguingly, we observed a statistically significant inverse correlation between *SPRY4* and the expression of EZH2 and MDM2 (Figure 4A–4C). Upon *SPRY4* overexpression, the expression of EZH2 and MDM2 in SW480 were downregulated, which were similar with the treatment of GSK126 at 50 μM, indicating an underlying inhibition role of *SPRY4* towards EZH2 and MDM2. Next, the immunoprecipitation assay was used to evaluate the association between EZH2 and MDM2, and western blot was used to measure the expression of EZH2 and MDM2. We found that EZH2 and MDM2 could bind to each other (Figure 4D, 4E). Thus, taking the important role of p53 into consideration, the functional role of SPRY4 in CRC might be achieved partly through EZH2/MDM2/p53 axis.

### The effects of *SPRY4* silencing on proliferation, invasion and apoptosis were partly via regulating EZH2

**Figure 5:**
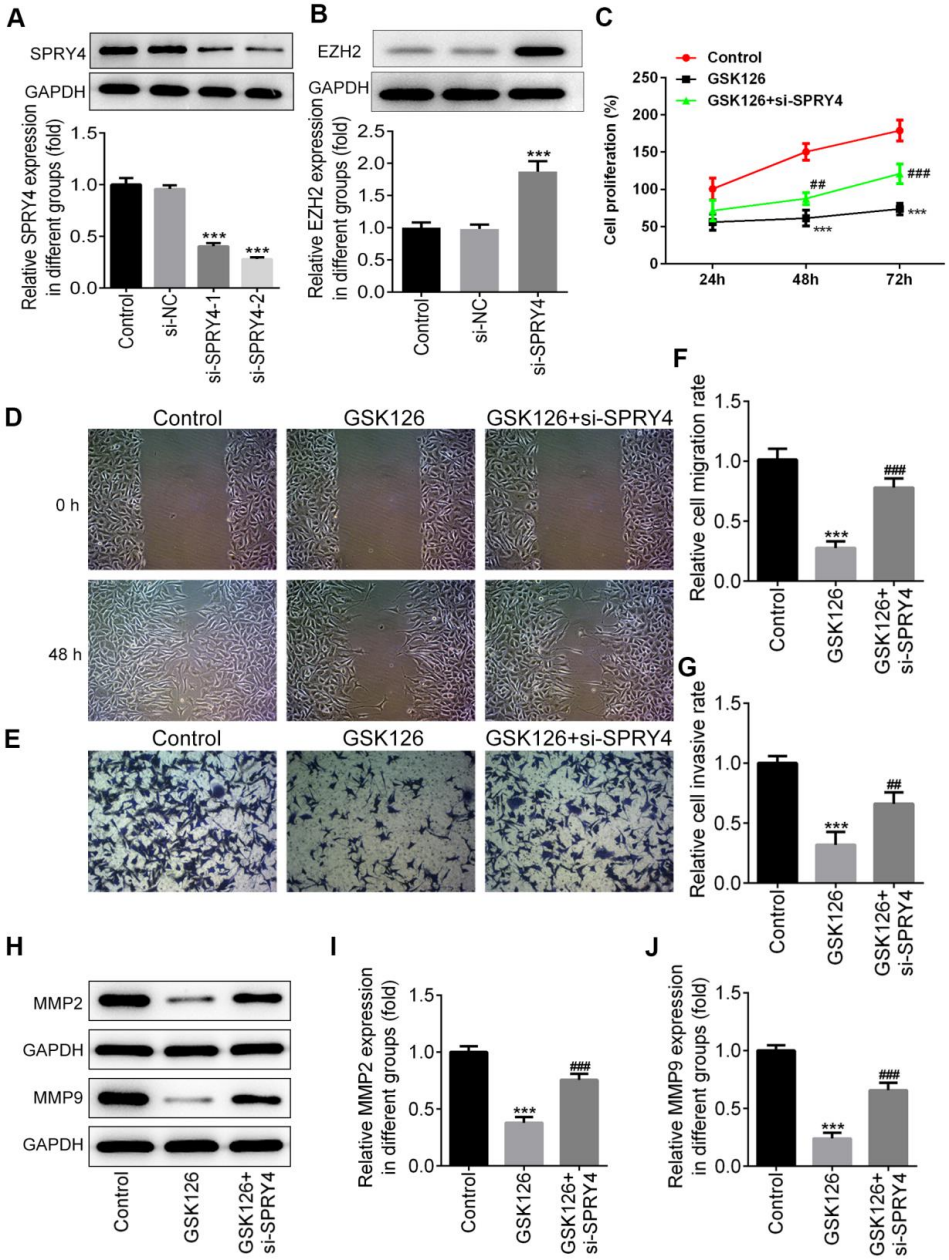
**The function of *SPRY4* silence on proliferation, migration and invasion in SW480 cells.** SW480 cells were transfected with si-*SPRY4* plasmid-1/2 and the empty plasmid, then the protein expression of *SPRY4* in different groups was measured using western blotting (**A**). After transfection, the protein expression of EZH2 was also measured using western blot (**B**). ***P<0.001 vs si-NC. SW480 cells were treated with GSK126 or co-treated with GSK126 and si-*SPRY4*. CCK-8 assay was conducted to determine cell proliferation ability (**C**). Wound-healing and Transwell assays were performed to determine cell migration and invasion abilities (**D**–**G**). The protein expression of MMP2 and MMP9 was measured using western blotting (**H**–**J**). ***p<0.001 vs Control; ##, ###p<0.01, 0.001 vs GSK126.

**Figure 6:**
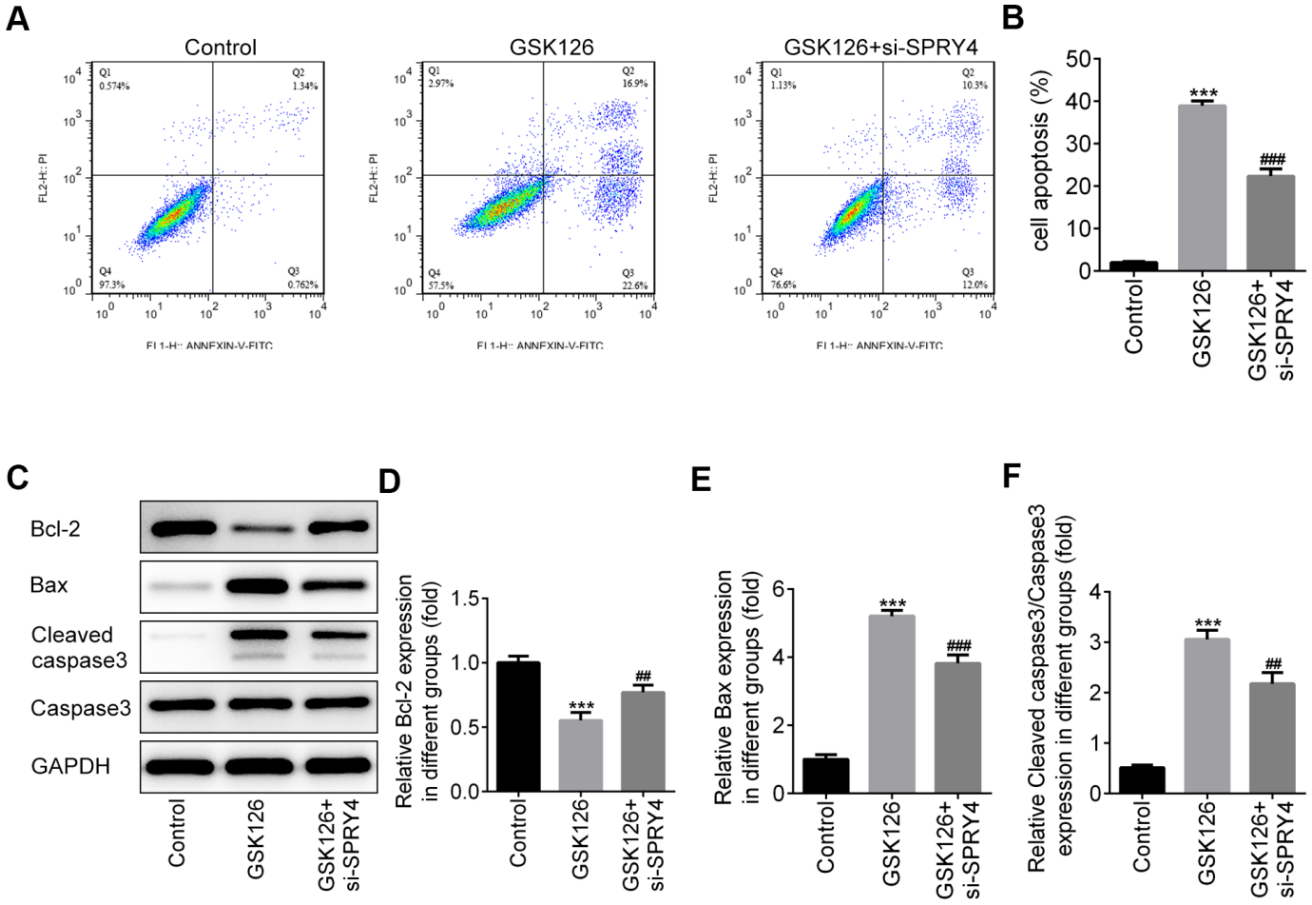
**The function of *SPRY4* silence on apoptosis in SW480 cells.** SW480 cells were treated with GSK126 or co-treated with GSK126 and si-SPRY4. Cell apoptotic rate was analyzed using flow cytometry assay (**A**, **B**). Moreover, the protein expressions of Bcl-2, Bax, Cleaved caspase-3 and caspase-3 were measured using western blotting (**C**–**F**). ***p<0.001 vs Control; ##, ###p<0.01, 0.001 vs GSK126.

To further study the impacts of *SPRY4*, we successfully transfected SW480 cells with si-SPRY4 plasmid (Figure 5A). The protein expression of EZH2 was demonstrated to be up-regulated after transfection with si-SPRY4 (Figure 5B). A series of cellular functional experiments showed that after *SPRY4* was knocked down, the proliferation of SW480 cells was greatly enhanced (reached ~200% after 72 h of incubation) and the inhibitory effects of GSK126 on cell proliferation in such cell were compromised. Similar trends were also observed in the wound-healing and Transwell assays. Compared with the GSK126 alone, GSK126 combined with si-SPRY4 transfection enhanced cell migration and invasion (Figure 5D–5G), upregulated expression of MMP2 and MMP9 (Figure 5H–5J), and reduced level of apoptosis (Figure 6A, 6B), as well as upregulated the expression of Bcl-2 and decreased the expression of Bax and cleaved caspase-3 (Figure 6C–6F). These results again highlighted the *SPRY4* as a repressor to oncogene *EZH2*.

### *SPRY4* regulated tumorigenesis *in vivo*

**Figure 7:**
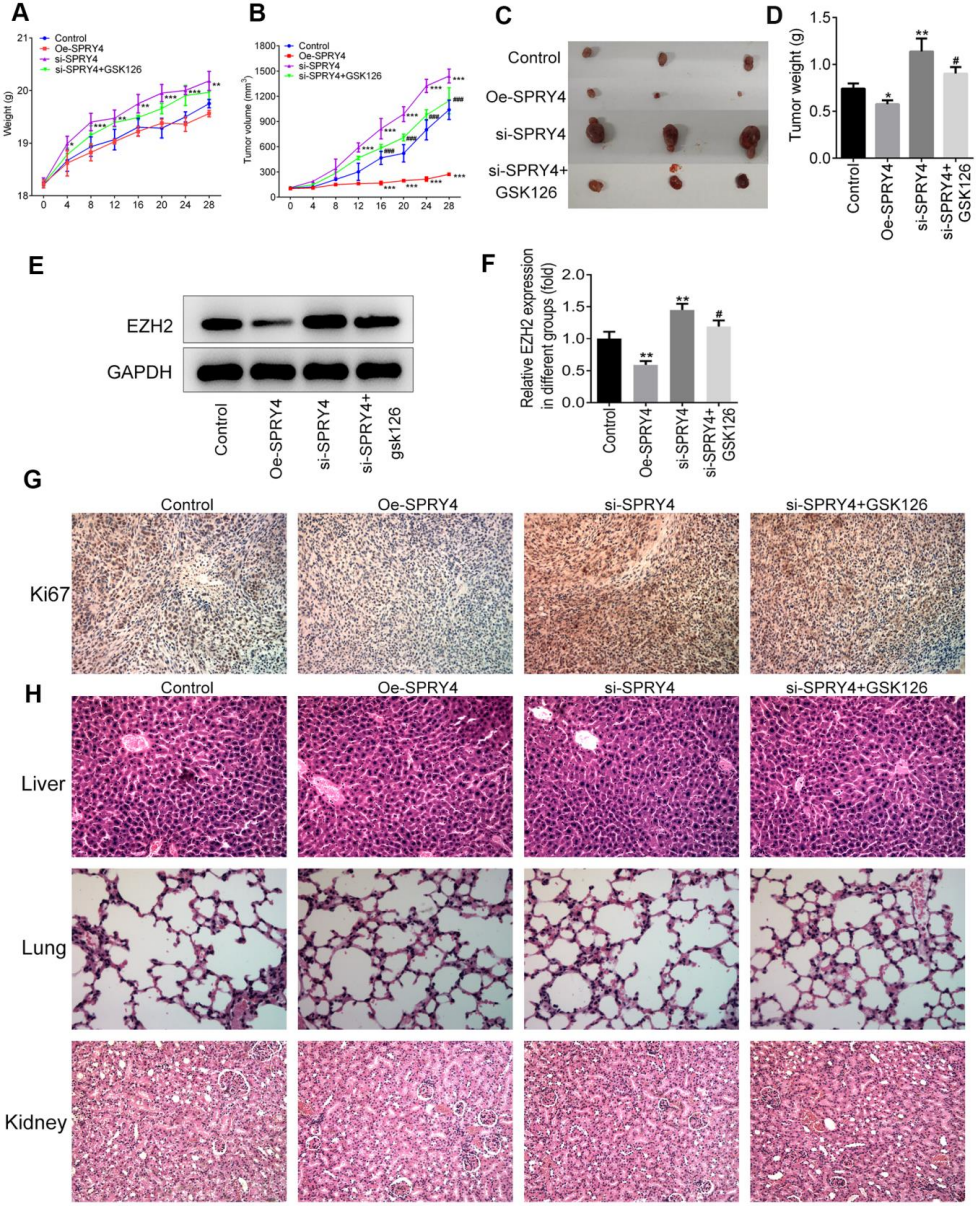
***SPRY4* regulated tumorigenesis *in vivo*.** The SW480 cells transfected with Oe-SPRY4 or si-SPRY4 were subcutaneously implanted into the axilla of the nude mice. The weight and tumor volume of mice were monitored every four days (**A**, **B**). After sacrifice, the total tumors were separated from the mice and were weighed (**C**, **D**). The protein expression of EZH2 in tumor tissues was detected using western blot (**E**, **F**). The expression of Ki67 was analyzed by immunohistochemistry (**G**). This histology of liver, lung and kidney was measured by H&E staining (**H**). *, **, ***p<0.05, 0.01, 0.001 vs Control; #, ###p<0.05, 0.001 vs si-*SPRY4*.

To further investigate the effects of *SPRY4* on tumorigenesis *in vivo*, we have additionally constructed plasmids to overexpress *SPRY4* (Oe-*SPRY4*) or silence *SPRY4* (si-*SPRY4*), respectively. The SW480 cells transfected with Oe-*SPRY4* or si-*SPRY4* were subcutaneously implanted into the axilla of the nude mice. The weight of mice and the tumor volume were monitored before sacrifice. Our results showed that both tumor size and tumor weight were reduced by Oe-*SPRY4* and increased by si-*SPRY4*, especially, and the promotive effect of si-*SPRY4* on tumor size and tumor weight was partly reversed by GSK126 (Figure 7A–7D). The protein expression of EZH2 in tumor tissues was reduced in Oe-SPRY4 group but increased in si-SPRY4 group (Figure 7E, 7F), indicating the involvement of EZH2 in the antitumor activity of *SPRY4*. Immunohistochemistry assay indicated that the expression of Ki67 was remarkably increased upon si-*SPRY4*, which was also partly declined upon co-treatment of si-*SPRY4* and GSK126 (Figure 7G). Moreover, H&E staining was applied to observe the morphological changes in major organs in different groups. As shown in Figure 7H, the liver, lung and kidney were not injured in each group, indicating that cancer metastasis did not occur. Collectively, our data demonstrated that the *SPRY4* regulated tumorigenesis *in vivo*.

## DISCUSSION

Pathogenesis and metastasis of CRC generally coincides with the successive dysregulation of certain genes. Even though mounting evidence has highlighted the *SPRY4* functions as tumor suppressor, the effects and the mechanism of *SPRY4* in regulating CRC progression remain unclear. The purpose of this study is to clarify whether the suppressive role of *SPRY4* in tumorigenesis involves modulation of CRC. In order to assess the impacts of *SPRY4* on CRC, we first measured the mRNA and protein expression of *SPRY4* in CRC cell lines and the normal colorectal cell line. We demonstrated that differential expression modality between cancer cells and normal cells in *SPRY4*, which hinted that *SPRY4* might serve as a tumor suppressor gene that negatively regulates tumor development.

Then, to clarify the role of *SPRY4* in CRC, we explored the effects of *SPRY4* on CRC cellular biological activities. The uncontrolled proliferation of CRC cells is the main cause for tumor metastasis. Recently, much attention has been paid on the exploration of specific genes or drugs which have inhibitory effects on cell proliferation during the development of CRC. For example, high expression of ubiquitin-like modifier-activating enzyme 2 (UBA2) was reported to be associated with poor prognosis of patients with CRC. Further basic experiments exhibited that downregulated UBA2 could significantly inhibit the proliferation of CRC cell lines *in vitro* and suppress tumor growth *in vivo* [25], and butyrate could interrupt the metabolism of CRC cells and ultimately result in the suppression of cell proliferation by targeting pyruvate kinase M2, thus suppressing the development of CRC [26]. These existing evidence revealed that inhibition of cell over-proliferation is an alternative approach to discover the effective treatment for CRC therapy. In the present study, we found that the overexpression of *SPRY4* significantly reduced cell proliferation by inhibiting cell viability and hindering colony formation of SW480 cells.

Apoptosis, as a process of the programmed cell death, is essential for the development and for the well-functioning of multicellular systems, and also is an important mechanism among anti-cancer research [27]. In this study, we found that overexpression of *SPRY4* significantly promoted cell apoptosis rate of SW480 cells. What’s more, overexpression of *SPRY4* showed significant inhibitory effects on the expression of Bcl-2 and promotive effects on the expression of Bax and cleaved caspase-3. Caspase-3 is known as an executioner caspase, and exerts critical effects during cell apoptosis by cleaving many key cellular proteins, no matter through intrinsic mitochondrial pathway or the extrinsic death receptor pathway [28]. Bcl-2 is a critical anti-apoptotic gene which can inhibit cell apoptosis by reducing the generation of reactive oxygen and hindering the release of cytochrome C. While Bax is regarded as a pro-apoptotic gene as the anti-apoptotic activity of Bcl-2 can be abolished by Bax [29]. Thus, these results suggested that the overexpression of *SPRY4* might promote apoptosis in SW480 cells by upregulating Bax and caspase-3 while downregulating Bcl-2.

We next investigated the possible targets of *SPRY4* in CRC cells through analysis by immunoprecipitation and western blotting. Considering the important role of p53 for SPRY4 and the close relationship among p53, MDM2 and EZH2, it is interesting to find that EZH2 is an appropriate theoretical candidate target of *SPRY4*. EZH2 is highly expressed in various malignant tumors and is closely involved the occurrence and development of cancers. EZH2 activation can promote expansion of breast tumor initiating cells [30], whereas GSK126, the novel EZH2 inhibitor, exerts anticancer effects on gastric cancer and prostate cancer by inhibiting cell migration, invasion and angiogenesis [31, 32]. In the present study, both the expression of EZH2 and MDM2 were downregulated upon *SPRY4* overexpression. Moreover, with the observation that GSK126 could mimic the inhibitory effect of *SPRY4* on EZH2 and MDM2, our work indicated that EZH2 might be a direct target of *SPRY4* in CRC. EZH2 was verified to bind to MDM2, and MDM2 could bind p53 to promote p53 degradation, which then influenced CRC progression [33, 34]. Thus, the antitumor effects of *SPRY4* might be achieved partly by regulating EZH2-mediated MDM2/p53 pathway. The more detailed information concerning on an in-depth research of the mechanism action of SPRY4 is deserved to be investigated in the future.

Collectively, this study found that *SPRY4* might act as an anti-tumor gene in CRC and *SPRY4* exerted a suppressive effect on CRC progression via the inhibition of EZH2. This study suggests that *SPRY4* may serve as a promising candidate of therapeutic targets for CRC treatment.

## MATERIALS AND METHODS

### Cells

Human colorectal epithelial cell line NCM460 and colorectal cancer cell lines, including SW620, SW480, LOVO and HCT116, were obtained from American Type Culture Collection (ATCC, Manassas, VA, USA). All cells were cultured in complete Dulbecco’s modified Eagle medium (DMEM; HyClone, Logan, UT) with 10 % fetal bovine serum (FBS; HyClone, Logan, UT).

### Quantitative real-time PCR

Trizol reagent (Beyotime, Haimen, China) was applied to extract entire RNA. After determining the quality and concentration of the extracted RNA, 1 μg of RNA was reverse transcribed to complementary DNA (cDNA) by a reverse transcriptase (TaKaRa, Japan). Then, quantitative real time PCR (qRT-PCR) was conducted using the SYBR-Green method (Applied Biosystems, USA) to detect the expression level of corresponding genes following the manufacturer’s guide. The primers used were listed as follows: *SPRY4* (forward-5′-TCTGACCAACGGCTCTTAGAC-3′, reverse- 5′-GTGCCATAGTTGACCAGAGTC-3′), *EZH2* (forward 5′-TTGTTGGCGGAAGCGTGTAAAATC-3′, reverse-5′-TCCCTAGTCCCGCGCAATGAGC-3′), and *β-actin* (forward-5′-GCACCACACCTTCTACAATG-3′, reverse-5′-TGCTTGCTGATCCACATCTG). RT-qPCR was performed in triplicate for each sample. The mRNA level of these genes was calculated using 2^-ΔΔCT^ method and normalized to that of β-actin mRNA.

### Western blotting

Total protein was extracted from the cells using lysis buffer containing proteinase inhibitor cocktail. The protein concentration was measured using the BCA protein assay kit (Beyotime, Haimen, China). Then, the same amount of protein was resolved by 12% SDS-PAGE and transferred to PVDF membranes, followed by blocking of 5 % skimmed milk for 2 h at room temperature. Subsequently, the membranes were incubated with primary antibodies at 4° C overnight. After horseradish peroxidase (HRP)-conjugated second incubation, the bands were developed by ECL assay (Beyotime, Haimen, China). The antibodies used were as followed: Anti-SPRY4 antibody (ab59785), anti-KMT6/EZH2 antibody (ab186006), anti-MDM2 antibody (ab38618), anti-Bcl-2 antibody (ab182858), anti-Bax antibody (ab32503), anti-MMP2 antibody (ab215986), anti-MMP9 antibody (ab219372), anti-Cleaved Caspase-3 antibody (ab32042), anti-β-actin antibody (ab8227) and Rabbit IgG, monoclonal- Isotype Control (ab172730) were obtained from Abcam (MA, USA).

### Cell transfection

Cell transfection was conducted using lipofectamine 2000 (Invitrogen, USA) in accordance with the guide of the manufacturer. The pcDNA3.1-*SPRY4* (Oe-SRPY4) and pcDNA3.1 negative control (Oe-NC) vectors were obtained from GenePharma (Shanghai, China). SW480 cells were transfected with 50 nM Oe-SRPY4 and Oe-NC, respectively. 48 after transfection, the transfection efficacy was detected by qRT-PCR and western blotting.

### Cell proliferation assay

Cell proliferation was determined using Cell Counting Kit-8 (CCK-8) assay. In brief, the transfected SW480 cells were inoculated into 96-well plates (2000 cells/well) and incubated in a humidified atmosphere with 5% CO_2_ at 37° C. After incubation for various durations, 10 μl of CCK-8 agent was added into each well for a further 3 h’s incubation. Afterwards, a microplate reader (Bio-Tek, USA) was used to detect the optical density of each well at 450 nm.

### Colony formation assay

The transfected SW480 cells were seeded into 6-well plates (400 cells/well) and incubated for 2 weeks. During this period, culture medium was replaced every three days. At last, cells were fixed with 4 % paraformaldehyde and stained with 0.1 % crystal violet to observe the colony formation.

### EdU assay

The transfected SW480 cells were inoculated into 24-well plates and cultured in a humidified atmosphere with 5% CO_2_ at 37° C for 24 h. EdU (50 μmol/L) was added for a further incubation of 8 h. Afterwards, cells were fixed with 4% formaldehyde at room temperature for 15 min, followed by incubation with Triton X-100 for 15 min. Subsequently, cells were incubated with EdU agent and stained with DAPI. Finally, cells were observed under a confocal microscopy (Olympus, Japan).

### Wound-healing assay

The transfected SW480 cells were seeded into 6-well plates. When 100% confluence achieved, a scratch was generated by using a 20 μl pipette tip. Then, the culture medium was replaced to DMEM without FBS. Cell images were obtained at 0 and 48 h under a light microscope (Olympus, Japan).

### Transwell assay

The transfected SW480 cells suspended in FBS-free DMEM were inoculated into the upper chamber of a 24-well Transwell pre-coated with Matrigel (BD Biosciences, USA) at a density of 4 x 10^4^ cells per well. The complete medium with 10% FBS was added to the lower chamber of the Transwell. 48 h later, the cells on the surface of the upper chamber were wiped out. The invasive cells were fixed with 4 % paraformaldehyde and were stained with 0.1 % crystal violet. The images were visualized using a light microscope (Olympus, Japan).

### Flow cytometry analysis

The transfected SW480 cells were collected, washed with cold PBS, and re-suspended in 100 μL of binding buffer. Subsequently, cells were stained with annexin V-FITC and propidium iodide (PI) according to the guide of the manufacturer. The signal was acquired by a FACS caliber flow cytometry and analyzed with FACS Diva software (BD Biosciences, USA).

### *In vivo* tumor xenograft study

Male Balb/c nude mice at the age of 4-6 weeks were obtained from Shanghai SLAC laboratory animal co. Ltd (Shanghai, China). The animal experiments were carried out in accordance with the protocols approved by the Institutional Animal Care and Use Committee of The Affiliated Hospital of Kunming Medical University. SW480 cells were transfected with *SPRY4* knockdown plasmid or *SPRY4* overexpression plasmid. The transfected cells were subcutaneously injected into the axilla of the mice. GSK126 (an inhibitor of EZH2, Selleck) was administrated intravenously at a dose of 150 mg/kg. The tumor volume was calculated as follows: tumor volume = 1/2 × length ×width^2^. After 28 days, the mice were euthanized, and the tumors were weighed and collected for further tests.

### Hematoxylin-Eosin (H&E) staining

The tumor tissue samples were fixed in 4 % paraformaldehyde, dehydrated in ethanol and embedded in paraffin blocks. Then, the samples were cut into 5-μm-thickness of sections. After staining with H&E reagent, the images were observed under a light microscope for randomly five fields.

### Immunohistochemistry

The paraffin-embedded tissue samples were deparaffinized, rehydrated and subjected to antigen retrieval. After washing with PBS and blocked with PBS containing 5% normal goat serum, the sections were washed with PBS and incubated with primary antibody against Ki67 and biotinylated secondary antibody (Santa Cruz, CA, USA). The sections were developed with DAB staining and hematoxylin counterstain. All images were photographed for five randomly fields.

### Statistical analysis

Data were presented as mean ± SEM. All the data were analyzed using the SPSS 18.0 statistical software. The differences among groups were determined using one way ANOVA analysis followed by Tukey’s post hoc test. *p* < 0.05 was considered to indicate a statistically significant difference.
